# Supplementary material for: Spodoptera frugiperda transcriptional response to infestation by Steinernema carpocapsae
Source: Sci Rep. 2019 Sep 9;9:12879. doi: 10.1038/s41598-019-49410-8 (PMC6733877; doi:10.1038/s41598-019-49410-8)
Supplement: Supplementary file 1 — Supplementary information [file 41598_2019_49410_MOESM1_ESM.pdf]

**Supplementary informations**

*Spodoptera frugiperda* transcriptional response to infestation by *Steinernema carpocapsae*

Louise Huot[1], Simon George[1], Pierre-Alain Girard[1], Dany Severac[2], Nicolas Nègre[1,*] and Bernard Duvic[1,*]

[1] DGIMI, Univ Montpellier, INRA, Montpellier, France

[2] MGX, Univ Montpellier, CNRS, INSERM, Montpellier, France

[*] Co-corresponding authors

E-mail: nicolas.negre@umontpellier.fr (NN) ; bernard.duvic@umontpellier.fr (BD)

**Supplementary Table S1: RNAseq statistics**

**Supplementary Table S2: Primers sequences and genes investigated in qPCR**

**Supplementary Data S1: Differentially Expressed (DE) genes at 15 hpi**

This table presents the genes that are found differentially expressed upon EPN infestation at 15 hpi. The baseMean column represents the DESeq2 normalized mean expression levels of a particular gene across all experiments. The log2FoldChange and padj columns provided are given by the DESeq2 'results' command from pair-wise comparisons between (Midgut: MG, Fat Body: FB, Hemocytes: HC). On the right hand of the table, we present the best blastp homolog of each gene as well as our manual annotation of each gene based on this homology and protein domain analyses or on previous annotation [38] (in bold). Genes have been grouped by tissues where the differential expression false discovery rate (padj) was less than 0.01.

**Supplementary Data S2: Script used for the analysis of differential expression in the fat body at 8 hpi.**

**Supplementary Data S3: Script for global analysis of the EPN effect.**

**Supplementary Data S4: Heatmaps were generated using the heatmap.2 function of the gplots R package such as below that generated Fig. 1D.**

**Supplementary Figure S1: Dataset quality control**

**A:** Heatmap of pair-wise correlation between all RNAseq samples. Hierarchical clustering of samples shows the grouping of experiments mostly by tissues then by time-point and by condition. **B:** Principal Component analysis of RNAseq samples showing the grouping of experiments mostly by tissue then by time-point and by condition.

**Supplementary Figure S2: DESeq2 analysis**

MA plots showing the log2FoldChange in function of mean expression (measured in reads coverage) of total *S. frugiperda* transcripts in a pairwise DESeq2 analysis of EPN vs PBS in every Tissue*Time-point conditions. Indicated on each plot is the number of genes significantly overexpressed (red box) or under-expressed (blue box) upon EPN infestation. For experiments at 15 hpi, the total number of DE genes is also indicated on the right side of the plot.

**Supplementary Figure S3: DE genes in fat body at 8 hpi**

At 8 hpi, 5 genes are significantly differentially expressed in the fat body (FB08). This heatmap of log2FoldChange shows that the 4 unknown genes significantly overexpressed in the FB08 condition are also overexpressed at 15 hpi in all 3 tissues. On the right hand side, this table indicates the DESeq2 results for these genes, showing that the 4 overexpressed genes are of unknown function and correspond to the gene cluster presented in **Fig. 5B**.

**Supplementary Figure S4: Validation of RNAseq data using quantitative RT-PCR**

Selected genes were analyzed by quantitative PCR using RNA samples from tissues of naïve or infected larvae. The relative expression level (ratio infected/naïve larvae) is shown as log2FoldChange mean from 3 independent experiments.

**Supplementary Figure S5: Fat Body and Hemocytes Gene Ontology enrichment**

The DE genes common to FB and HC at 15 hpi (**Fig. 3C**) show an enrichment of Gene Ontology terms associated to immunity. The same analysis conducted on the other gene lists, in particular the 77 genes associated to HC15, did not produce significant GO term enrichments.

**Supplementary Figure S6: Structures and phylogeny of *Spodoptera frugiperda* peptidoglycan recognition proteins**

**A:** Structures of *Sf*PGRP. In red, signal peptide and in blue, transmembrane domain. **B:** The phylogeny of 105 PGRP domain amino acid sequences was determined by using the Maximum Likelihood method [96] in MEGA7 [97]. The bootstrap consensus tree was built from 1000 replicates and branches corresponding to partitions reproduced in less than 50% bootstrap replicates were collapsed. Initial tree(s) for the heuristic search were obtained automatically by applying Neighbor-Join and BioNJ algorithms to a matrix of pairwise distances estimated using a JTT model, and then selecting the topology with superior log likelihood value. Branches colors: green, Lepidopteran (Bm: *Bombyx mori*, Dp: *Danaus plexippus*, Hm: *Heliconius melpomene*, Ms: *Manduca sexta*, Px: *Papillio xuthus*, Sf: *Spodoptera frugiperda*), blue, Dipteran (Aa: *Aedes aegypti*, Ag: *Anopheles gambiae*, Dm: *Drosophila melanogaster*) and orange, Hymenopteran (Am: *Apis mellifera*, Nv: *Nasonia vitripennis*). *Manduca sexta* amino acid sequences were retrieved at ftp://ftp.bioinformatics.ksu.edu/pub/Manduca/OGS2/ [98]. *** Sequence from [21].

**Supplementary Figure S7: Phylogenetic analysis of genes from bacterial origin**

The 100 first best hits after blastp on nr NCBI were retrieved and the phylogenetic tree was constructed using the method described as in **Supplementary Fig**. **S6.** Sequences are grouped in two main clades, one with bacteria only and the second with Lepidoptera only.

**S1 Table**: RNAseq statistics

| Tissue | Condition | Sample | Total reads | Mapped reads | % Mapped reads |
|---|---|---|---|---|---|
| **Fat body** | Control 8 h | 3FBn8 | 29 360 077 | 16 556 358 | 56.4 |
| | | 5FBn8 | 28 766 960 | 16 431 320 | 57.1 |
| | Control 15 h | 1FBn15 | 8 160 217 | 5 760 726 | 70.6 |
| | | 3FBn15 | 31 182 081 | 21 405 010 | 68.7 |
| | | 5FBn15 | 101 295 028 | 67 865 194 | 67.0 |
| | Infected 8 h | 3FBi8 | 18 680 946 | 10 924 308 | 58.5 |
| | | 5FBi8 | 27 084 597 | 13 484 897 | 49.8 |
| | Infected 15 h | 1FBi15 | 38 001 141 | 22 705 073 | 59.8 |
| | | 3FBi15 | 25 680 693 | 15 233 458 | 59.3 |
| | | 5FBi15 | 17 018 902 | 9 757 977 | 57.3 |
| **Midgut** | Control 8 h | 1MGn8 | 40 762 942 | 26 432 615 | 64.8 |
| | | 3MGn8 | 37 701 095 | 24 444 804 | 64.8 |
| | | 5MGn8 | 39 108 039 | 23 913 146 | 61.2 |
| | Control 15 h | 1MGn15 | 53 104 319 | 33 125 675 | 62.4 |
| | | 5MGn15 | 56 673 439 | 35 116 354 | 62.0 |
| | Infected 8 h | 1MGi8 | 39 503 925 | 25 273 765 | 64.0 |
| | | 3 MGi8 | 45 389 710 | 29 365 492 | 64.7 |
| | | 5MGi8 | 33 930 844 | 21 504 374 | 63.4 |
| | Infected 15 h | 1MGi15 | 46 822 381 | 28 888 031 | 61.7 |
| | | 3MGi15 | 40 929 947 | 25 691 178 | 62.8 |
| | | 5MGi15 | 42 270 370 | 26 081 101 | 61.7 |
| **Hemocytes** | Control 15 h | 1HCn15 | 36 819 698 | 20 610 325 | 56.0 |
| | | 3HCn15 | 41 399 767 | 23 008 997 | 55.6 |
| | | 5HCn15 | 30 657 274 | 17 119 548 | 55.8 |
| | Infected 15 h | 1HCi15 | 32 486 507 | 18 101 920 | 55.7 |
| | | 3HCi15 | 73 456 056 | 40 045 769 | 54.5 |
| | | 5HCi15 | 33 712 769 | 18 309 356 | 54.3 |

*Samples abbreviations*: MG, midgut ; FB, fat body ; HC, hemocytes ; n, naive larvae ; i, infected larvae ; Number at the end (8 or 15) corresponds to the time of tissue extraction after infestation with nematodes.

**S2 Table**: Primers sequences and genes investigated in qPCR.

| Gene | Annotation | Primer | Sequence |
|---|---|---|---|
| GSSPFG00022277001-RA | TF Adf-1 | Adf1-F | CCGGAAGAGGAGGAAAAGTT |
| | | Adf1-R | CTAAGCGTTTGATGCGAGTG |
| GSSPFG00001145001.2-RA | Cactus | Cactus-F | ACTGACACACAGACCCCACA |
| | | Cactus-R | TCATCACAGCGAGGTGTAGC |
| GSSPFG00018355001.1-RA | Carboxy-peptidase D | CarboD-F | GGAGCGCATTGGTATTCTGT |
| | | CarboD-R | CGTTGCCTTCGATTTTGATT |
| GSSPFG00011909001.1-RA | Orf43(GB3) | GB3-F | GGTGTCGAGTTGGGAATCAT |
| | | GB3-R | TTCGTTGGAACATGGACTCA |
| GSSPFG00010207001-RA | Rab5 GEF | GEFRab5-F | ATCACCATCCAGCCAGAAAC |
| | | GEFRab5-R | GTCGGGAGTCATCAGTTCGT |
| GSSPFG00000179001.3-RA | GNBP3 | GNBP3-F | CCAAGGTGCCCTCATCTTTA |
| | | GNBP3-R | TGATGCATAGCTTCCGTCAG |
| GSSPFG00027637001-RA | IMPI(27637) | IMPI27637-F | TTGCAAGGAGATGCAGAATG |
| | | IMPI27637-R | TAGCGGTAGCCACAATTTGA |
| GSSPFG00005843001.1 | J-dom cont. protein | JDomProt-F | TGCAGTACCACCCTGACAAG |
| | | JDomProt-R | ATGCGATCCTTGGTGTTAGG |
| GSSPFG00023266001-RA | LITAF | LITAF-F | AACAGCAACACCAGCAACAG |
| | | LITAF-R | AAACAAAGCCCAAGAGCAAA |
| GSSPFG00006503001.4-RA | PGRP-S6 | PGRPS6-F | TCCCTACAACTTCCCATTCG |
| | | PGRPS6-R | CAGCCGTGGTCATCAATATG |
| GSSPFG00035420001.3-RA | Relish | Relish-F | TATGGCACCAACAAAACGAA |
| | | Relish-R | CGATACCACCGAACCTGACT |
| GSSPFG00007143001-RA | Sialin | Sialin-F | AGTCACCACCATTCCTTTCG |
| | | Sialin-R | CAGGGTCCTACTCAGGGTCA |
| GSSPFG00026933001-RA | Unk3 | UNK3-F | AGGAGACACCAATCGCAAAG |
| | | UNK3-R | CCAGCAGCAAGAATGTTGAA |
| GSSPFG00026935001-RA | Unk4 | UNK4-F | ACTAATGGGCTCCCTCCTGT |
| | | UNK4-R | GTAGTTGGGGATCCTGCTGA |
| GSSPFG00032366001-RA | Zonadhesin | Zona-F | TGCTTGCCTGGTTGTATCTG |
| | | Zona-R | CTGTGTGCATCTTCGCAACT |
| GSSPFG00020532001-RA | RpL32 | Rpl32-F | TACAATCGTCAAAAAGAGGACGA |
| | | Rpl32-R | AAACCATTGGGTAGCATGTG |
| GSSPFG00002766001-RA | EF1$\alpha$ | EF1a-F | GACGTATACAAAATCGGTGGTATT |
| | | EF1a-R | GATTTGATGGATTTAGGGTTGTCT |

**S2 Data**: Script used for the analysis of differential expression in the fat body at 8 hpi

```
#Importing read counts
> CG08.counts <- read.table("FB_08.counts.txt", header=T, row.names=1)
> head(CG08.counts)
                        FB08_PBS_1   FB08_PBS_2   FB08_PBS_3   FB08_NEP_1   FB08_NEP_2   FB08_NEP_3
GSSPFG00034586001-RA             0            0            0            0            0            0
GSSPFG00034587001-RA           256          294          222          232          165          201
GSSPFG00034588001-RA           209          204          137          104          101          145
GSSPFG00034589001-RA            79           71           39           39           35           49
GSSPFG00034590001-RA           148           77           43          187           66           39
GSSPFG00034591001-RA            16           11           15           15            7           15
#Assigning metadata to each sample
> FB08.meta <- read.table("FB_08_meta.txt", header=T, row.names=1)
> FB08.meta
                   Tissue    Condition         Time     Platform
FB08_PBS_1        FatBody          PBS          08H          MGX
FB08_PBS_2        FatBody          PBS          08H          MGX
FB08_PBS_3        FatBody          PBS          08H          MGX
FB08_NEP_1        FatBody          NEP          08H          MGX
FB08_NEP_2        FatBody          NEP          08H          MGX
FB08_NEP_3        FatBody          NEP          08H          MGX
#Creating a DESeq2 dataset object
> library(DESeq2)
> dds.FB08 <- DESeqDataSetFromMatrix(countData=CG08.counts,FB08.meta, design=~Condition)
>dds.FB08$Condition <- relevel(dds.FB08$Condition, ref = "PBS")
#Performing differential analysis expression
> dds.FB08 <- DESeq(dds.FB08)
> res.FB08 <- results(dds.FB08)
> res.FB08
log2 fold change (MAP): Condition PBS vs NEP
Wald test p-value: Condition PBS vs NEP
DataFrame with 21779 rows and 6 columns
                        BaseMean   log2FoldChange       lfcSE           stat         pvalue        padj
                       <numeric>        <numeric>   <numeric>      <numeric>      <numeric>   <numeric>
GSSPFG00034586001-RA     0.00000               NA          NA             NA             NA          NA
```

```
GSSPFG00034587001-RA   226.12105      -0.09167128    0.2563811     -0.3575587      0.7206736   0.9999365
GSSPFG00034588001-RA   146.89507       0.16170379    0.3390730      0.4768996      0.6334336   0.9999365
GSSPFG00034589001-RA    50.82056       0.12751588    0.4220970      0.3021009      0.7625752   0.9999365
GSSPFG00034590001-RA    92.22889      -0.48799757    0.5859347     -0.8328532      0.4049275   0.9999365
...                          ...              ...          ...            ...            ...         ...
GSSPFG00017764001-RA   0.1530537       0.08952471    0.7413174      0.1207644      0.9038777          NA
GSSPFG00002544001-RA   8.1074389       0.12156989    0.7613174      0.1596836      0.8731303   0.9999365
GSSPFG00002545001-RA  83.5576439       0.26476578    0.3550677      0.7456770      0.4558626   0.9999365
GSSPFG00002546001-RA   3.0554269       0.18693003    0.8997145      0.2077659      0.8354117   0.9999365
GSSPFG00002547001-RA   0.1383452       0.08952471    0.7413174      0.1207644      0.9038777          NA
> sum(res.FB08$padj < 0.01, na.rm=T)
[1] 5
#Generating MA plots
> plotMA(res.FB08, ylim=c(-10,10))
> quartz.save("MA_FB08.tiff", type="tiff", dpi=720)
> dev.off()
```

**S3 Data**: Script for global analysis of the NEP effect

```
> NEP.FULL.meta
                  Tissue     Condition Time       Platform
FB08_PBS_1         FatBody             PBS  08H            MGX
FB08_PBS_2         FatBody             PBS  08H            MGX
FB08_PBS_3         FatBody             PBS  08H            MGX
FB08_NEP_1         FatBody             NEP  08H            MGX
FB08_NEP_2         FatBody             NEP  08H            MGX
FB08_NEP_3         FatBody             NEP  08H            MGX
FB15_PBS_1         FatBody             PBS  15H           GATC
FB15_PBS_2         FatBody             PBS  15H           GATC
FB15_PBS_3         FatBody             PBS  15H           GATC
FB15_NEP_1         FatBody             NEP  15H            MGX
FB15_NEP_2         FatBody             NEP  15H            MGX
FB15_NEP_3         FatBody             NEP  15H            MGX
HC15_PBS_1       Hemocytes             PBS  15H           GATC
HC15_PBS_2       Hemocytes             PBS  15H           GATC
HC15_PBS_3       Hemocytes             PBS  15H           GATC
HC15_NEP_1       Hemocytes             NEP  15H           GATC
HC15_NEP_2.1     Hemocytes             NEP  15H           GATC
HC15_NEP_2.2     Hemocytes             NEP  15H           GATC
HC15_NEP_3       Hemocytes             NEP  15H           GATC
MG08_PBS_1          MidGut             PBS  08H            MGX
MG08_PBS_2          MidGut             PBS  08H            MGX
MG08_PBS_3          MidGut             PBS  08H            MGX
MG08_NEP_1          MidGut             NEP  08H            MGX
MG08_NEP_2          MidGut             NEP  08H            MGX
MG08_NEP_3          MidGut             NEP  08H            MGX
MG15_PBS_1          MidGut             PBS  15H           GATC
MG15_PBS_3          MidGut             PBS  15H           GATC
MG15_NEP_1          MidGut             NEP  15H            MGX
MG15_NEP_2          MidGut             NEP  15H            MGX
MG15_NEP_3          MidGut             NEP  15H            MGX
> dds.FullNEP <- DESeqDataSetFromMatrix(countData=Counts.allNEP, NEP.FULL.meta, design =
~Platform+Tissue+Time+Condition)
```

```
> design(dds.FullNEP) <- ~ Platform + Condition
> dds.FullNEP <- DESeq(dds.FullNEP)
> dds.FullNEP <- DESeq(dds.FullNEP, test="LRT", reduced=~Platform)
> res.NEP.LRT <- results(dds.FullNEP)
> res.NEP.LRT
log2 fold change (MLE): Condition PBS vs NEP
LRT p-value: '~ Platform + Condition' vs '~ Platform'
DataFrame with 21779 rows and 6 columns
                         baseMean   log2FoldChange       lfcSE          stat       pvalue       padj
                        <numeric>        <numeric>    <numeric     <numeric>    <numeric>  <numeric>
GSSPFG00034586001-RA      0.00000               NA          NA            NA           NA         NA
GSSPFG00034587001-RA    397.16106      -0.63382359   0.2725597   4.887725886   0.02704829  0.2803752
GSSPFG00034588001-RA    239.01600      -0.63090704   0.2599847   5.633063087   0.01762481  0.2365014
GSSPFG00034589001-RA     51.20187       0.02851923   0.4314123   0.004343166   0.94745529  0.9798495
GSSPFG00034590001-RA    215.78401       1.59117230   0.7122354   2.202168787   0.13781672  0.4972546
...                           ...              ...         ...           ...          ...        ...
GSSPFG00017764001-RA    0.5151814       -0.2097926   2.2026076     0.0395469    0.8423691         NA
GSSPFG00002544001-RA   23.8805964       -0.2642303   0.2908762     0.8449198    0.3579935  0.6863636
GSSPFG00002545001-RA   96.6551035       -0.3146891   0.2146926     2.2883293    0.1303502  0.4876959
GSSPFG00002546001-RA    2.8121936       -1.0511276   0.7431343     1.5893673    0.2074165  0.5660697
GSSPFG00002547001-RA    0.5012959        1.6010805   2.3033557     0.4597224    0.4977537         NA
> summary(res.NEP.LRT.Sig)
out of 271 with nonzero total read count
adjusted p-value < 0.1
LFC > 0 (up)      : 22, 8.1%
LFC < 0 (down)    : 249, 92%
outliers [1]      : 0, 0%
low counts [2]    : 0, 0%
```

**S4 Data**: Heatmaps were generated using the heatmap.2 function of the gplots R package such as below that generated **Fig 1D**

```
> LRTGenes.normcounts <- read.table("LRT_NEP_genes_normcounts", header=T, row.names=1)
> head(LRTGenes.normcounts)
                         FB08_PBS_1  FB08_PBS_2  FB08_PBS_3  FB08_NEP_1  FB08_NEP_2  FB08_NEP_3  FB15_PBS_1   FB15_PBS_2  FB15_PBS_3
GSSPFG00016231001-RA      128.32836   199.96122   257.85743   154.97011   258.37224   229.76859   129.77399    266.22693   251.08681
GSSPFG00032367001-RA      434.72475   377.93718   416.35695   422.32312   434.06536   348.40728   589.39020    640.86586   537.98631
GSSPFG00004566001-RA      425.77161   386.31251   542.92000   348.97849   492.62974   887.53751   805.68018   1114.31066   840.00673
GSSPFG00021577001.3-RA  251.68275   203.10197    16.55965    36.67232    48.22948    21.02458   156.81024     63.12597   101.86723
GSSPFG00006115001.1-RA   44.76571    42.92361    69.78710    36.67232    24.11474    69.08075   113.55224    104.29509    64.86078
GSSPFG00017500001-RA       65.65637    70.14346    67.42144    36.67232   115.40627    87.10182    37.85075     71.35980    33.42518
                         FB15_NEP_1  FB15_NEP_2  FB15_NEP_3  HC15_PBS_1  HC15_PBS_2  HC15_PBS_3  HC15_NEP_1 HC15_NEP_2.1
GSSPFG00016231001-RA       418.8384    266.2224    282.8820   600.61412   201.39679   470.80267    468.7684     698.9269
GSSPFG00032367001-RA       256.5578    401.3204    386.4371   639.86995   685.26384   827.54095    468.7684     426.9121
GSSPFG00004566001-RA      2282.7466    731.1183    914.3152  1265.34610  1270.15100  1969.71587   3454.7344    6986.7507
GSSPFG00021577001.3-RA   5457.2635    162.9122    202.0586   146.55508   137.05277    97.22266   1734.4429    3169.7281
GSSPFG00006115001.1-RA    129.8245    123.1775    103.5550    30.75040    30.24169    29.09025    328.1379     702.7049
GSSPFG00017500001-RA       143.7342    176.8194    191.9557    12.43101    12.22536    13.01406    107.9052     222.9010
                       HC15_NEP_2.2  HC15_NEP_3  MG08_PBS_1  MG08_PBS_2  MG08_PBS_3  MG08_NEP_1  MG08_NEP_2   MG08_NEP_3  MG15_PBS_1
GSSPFG00016231001-RA       796.4090   593.33708   174.79963   269.15742  187.500895   209.51486   274.24550   221.835168   241.27936
GSSPFG00032367001-RA       457.0192   397.22708   494.66119   425.11040  404.882411   430.78468   354.78847   436.086228   550.76849
GSSPFG00004566001-RA      7118.8299  5731.41923   753.59675   716.43372  892.683545   752.31738   793.44814   813.395616  3053.15104
GSSPFG00021577001.3-RA   3181.7795  1566.37651    36.99079    30.08230    7.470155    24.20139    15.97547     1.896027    33.59586
GSSPFG00006115001.1-RA    717.9894   317.11405    44.24389    42.74853   50.050040    44.94543    34.61351    31.284447    32.57780
GSSPFG00017500001-RA       210.1903    99.30677    58.75008    58.58132   66.484381    52.55158    27.95707    45.504650    28.50558
                         MG15_PBS_3  MG15_NEP_1  MG15_NEP_2  MG15_NEP_3
GSSPFG00016231001-RA     334.154738    291.7330   288.23084   250.52902
GSSPFG00032367001-RA     683.271629    375.2607   447.19451   322.35304
GSSPFG00004566001-RA    1107.199282   1240.6329  1187.86043  1113.27231
GSSPFG00021577001.3-RA    1.870269    492.5681    34.93707   111.15622
GSSPFG00006115001.1-RA   42.392765    128.9767    18.34196    33.34687
GSSPFG00017500001-RA      28.677459    137.5751    23.58252    34.20191
> library(gplots)
> heatmap.2(as.matrix(LRTGenes.normcounts), Rowv=T, Colv=NA, dendrogram="none", cexRow=0.1,key=FALSE,
keysize= 0.75, symkey=FALSE, density.info="none", col=colorpanel(99, low="skyblue", mid="white",
high="red"), scale="row", colsep = c(3,6,9,12,15,19,22,25,27), sepcolor="black",labRow = NULL,
trace="none", margins = c(7,7), na.color="lightgray")
```

S1 Fig

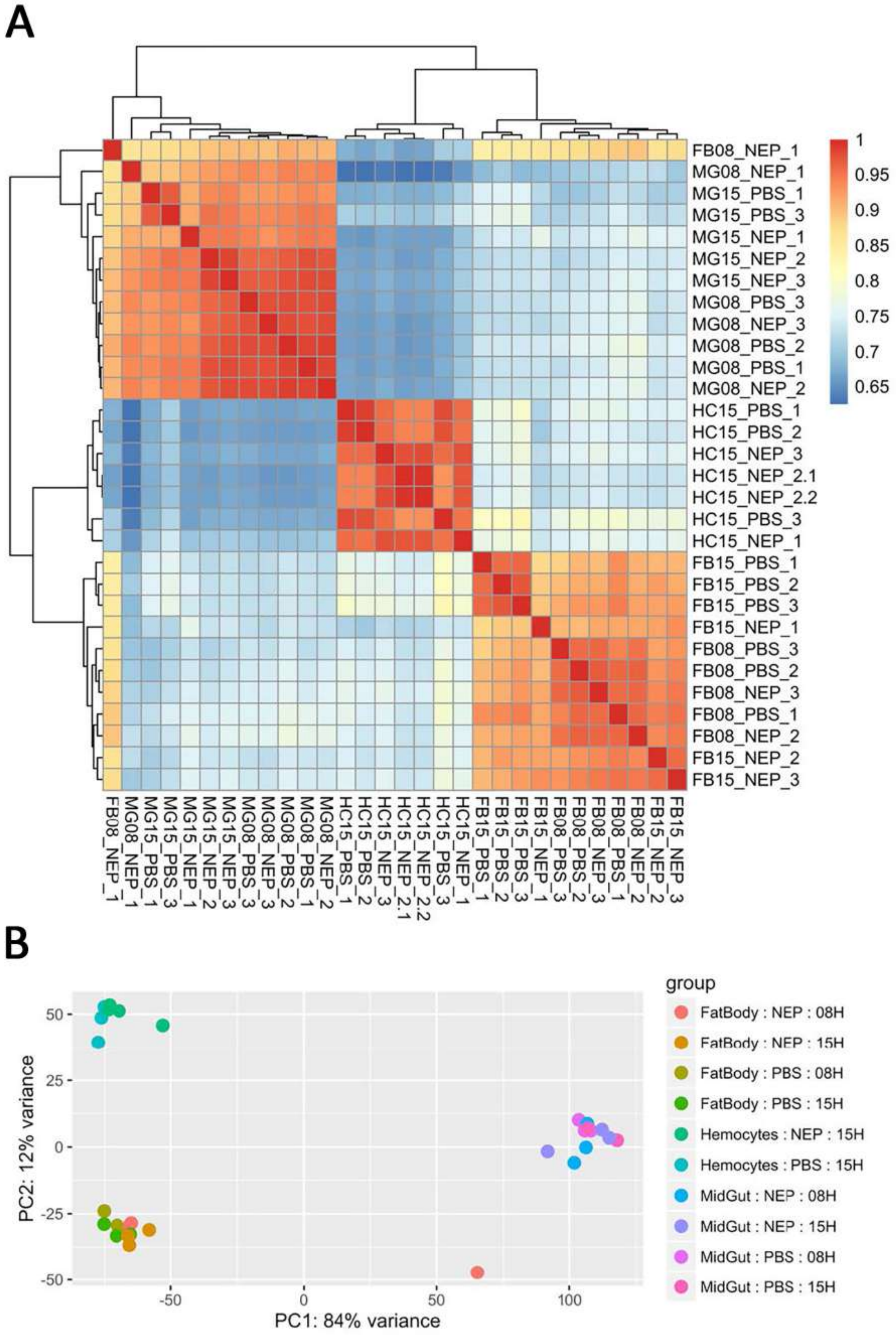
A
FB08_NEP_1
MG08_NEP_1
MG15_PBS_1
MG15_PBS_3
MG15_NEP_1
MG15_NEP_2
MG15_NEP_3
MG08_PBS_3
MG08_NEP_3
MG08_PBS_2
MG08_PBS_1
MG08_NEP_2
HC15_PBS_1
HC15_PBS_2
HC15_NEP_3
HC15_NEP_2.1
HC15_NEP_2.2
HC15_PBS_3
HC15_NEP_1
FB15_PBS_1
FB15_PBS_2
FB15_PBS_3
FB15_NEP_1
FB08_PBS_3
FB08_PBS_2
FB08_NEP_3
FB08_PBS_1
FB08_NEP_2
FB15_NEP_2
FB15_NEP_3
1
0.95
0.9
0.85
0.8
0.75
0.7
0.65
B
group
FatBody : NEP : 08H
FatBody : NEP : 15H
FatBody : PBS : 08H
FatBody : PBS : 15H
Hemocytes : NEP : 15H
Hemocytes : PBS : 15H
MidGut : NEP : 08H
MidGut : NEP : 15H
MidGut : PBS : 08H
MidGut : PBS : 15H
PC2: 12% variance
PC1: 84% variance
50
25
0
-25
-50
-50
0
50
100

S2 Fig

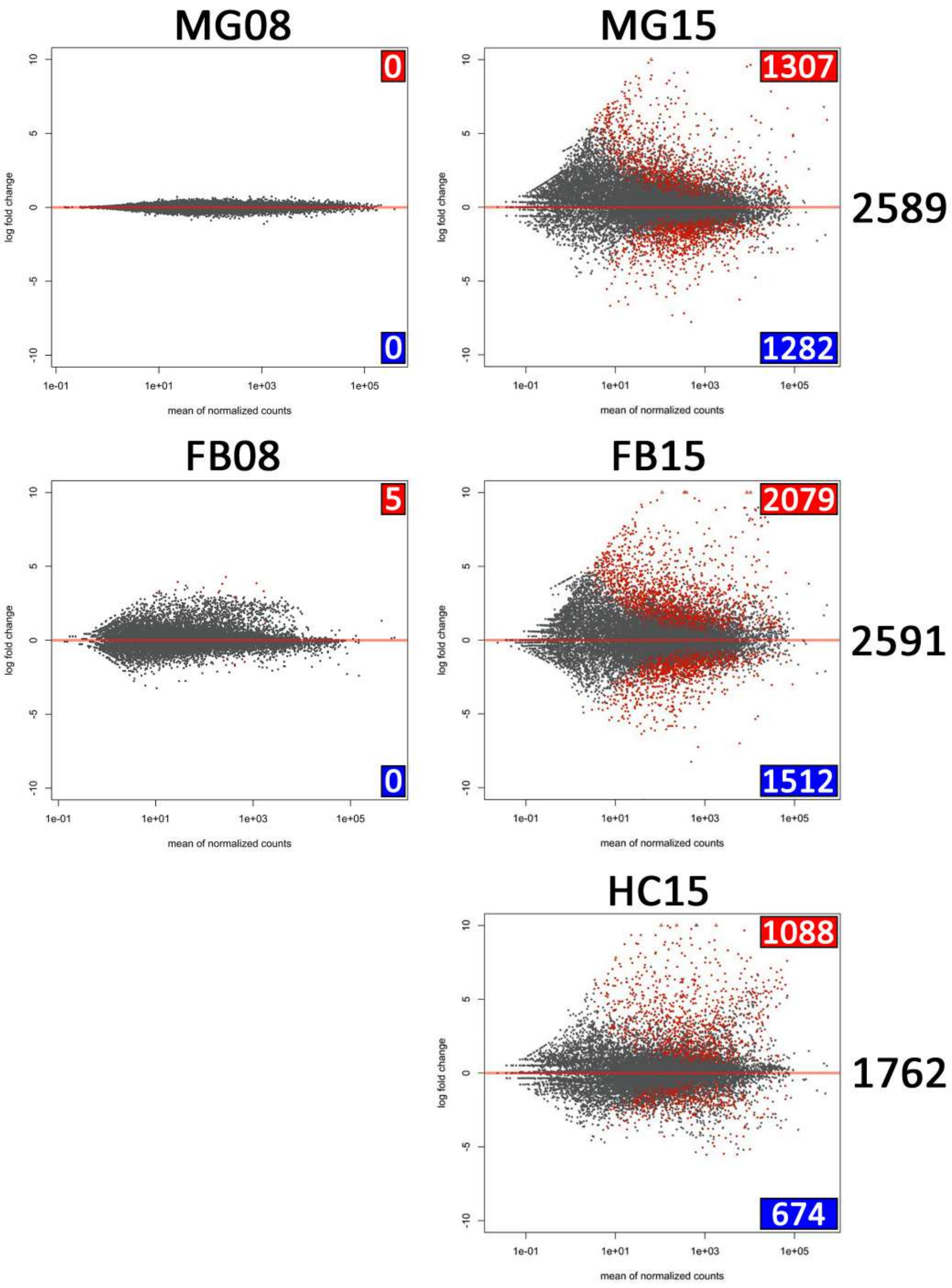
MG08
0
0
MG15
1307
1282
2589
FB08
5
0
FB15
2079
1512
2591
HC15
1088
674
1762
log fold change
mean of normalized counts
1e-01
1e+01
1e+03
1e+05
10
5
0
-5
-10

S3 Fig

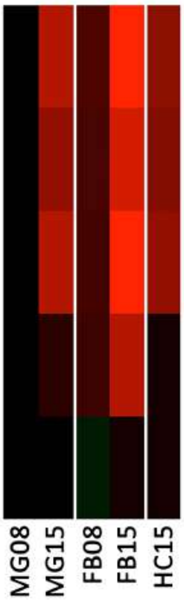


| | basemean | log2FoldChange | padj | scaffold | Start | End | Strand | Annotation |
|---|---|---|---|---|---|---|---|---|
| GSSPFG00022683001-RA | 233.4293865 | 3.814191758 | 4.30E-10 | scaffold_2226 | 14026 | 15443 | MINUS | Unknown |
| GSSPFG00026935001-RA | 276.4705843 | 4.280350878 | 4.30E-10 | scaffold_520 | 56730 | 58368 | MINUS | Unknown |
| GSSPFG00026933001-RA | 1178.098336 | 3.851037315 | 2.28-E07 | scaffold_520 | 44147 | 46056 | PLUS | Unknown |
| GSSPFG00030863001-RA | 1652.960416 | 3.321135688 | 4.02-E04 | scaffold_2502 | 26851 | 28889 | PLUS | Unknown |
| GSSPFG00010839001-RA | 672.3847102 | -1.46455364 | 8.98-E03 | scaffold_466 | 64317 | 77578 | MINUS | phosphoenolpyruvate carboxykinase |

## S4 Fig

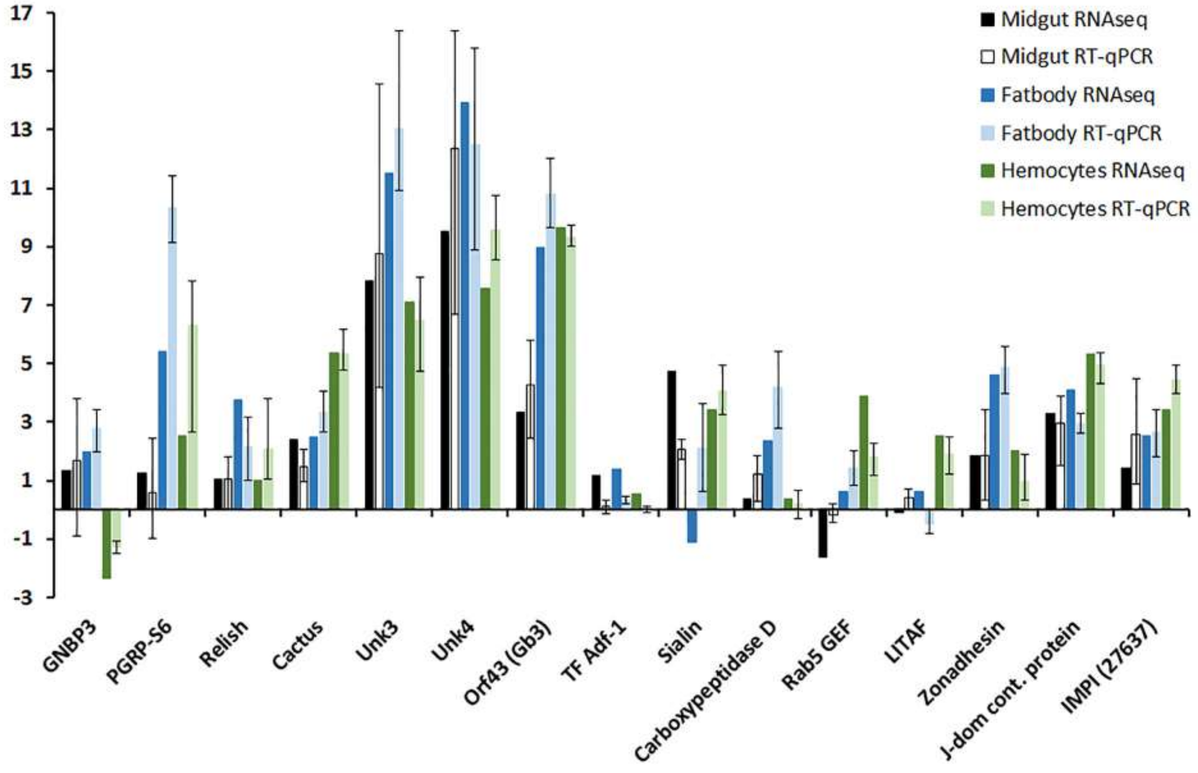

17
15
13
11
9
7
5
3
1
-1
-3
Midgut RNAseq
Midgut RT-qPCR
Fatbody RNAseq
Fatbody RT-qPCR
Hemocytes RNAseq
Hemocytes RT-qPCR
GNBP3
PGRP-S6
Relish
Cactus
Unk3
Unk4
Orf43 (Gb3)
TF Adf-1
Sialin
Carboxypeptidase D
Rab5 GEF
LITAF
Zonadhesin
J-dom cont. protein
IMPI (27637)

S5 Fig

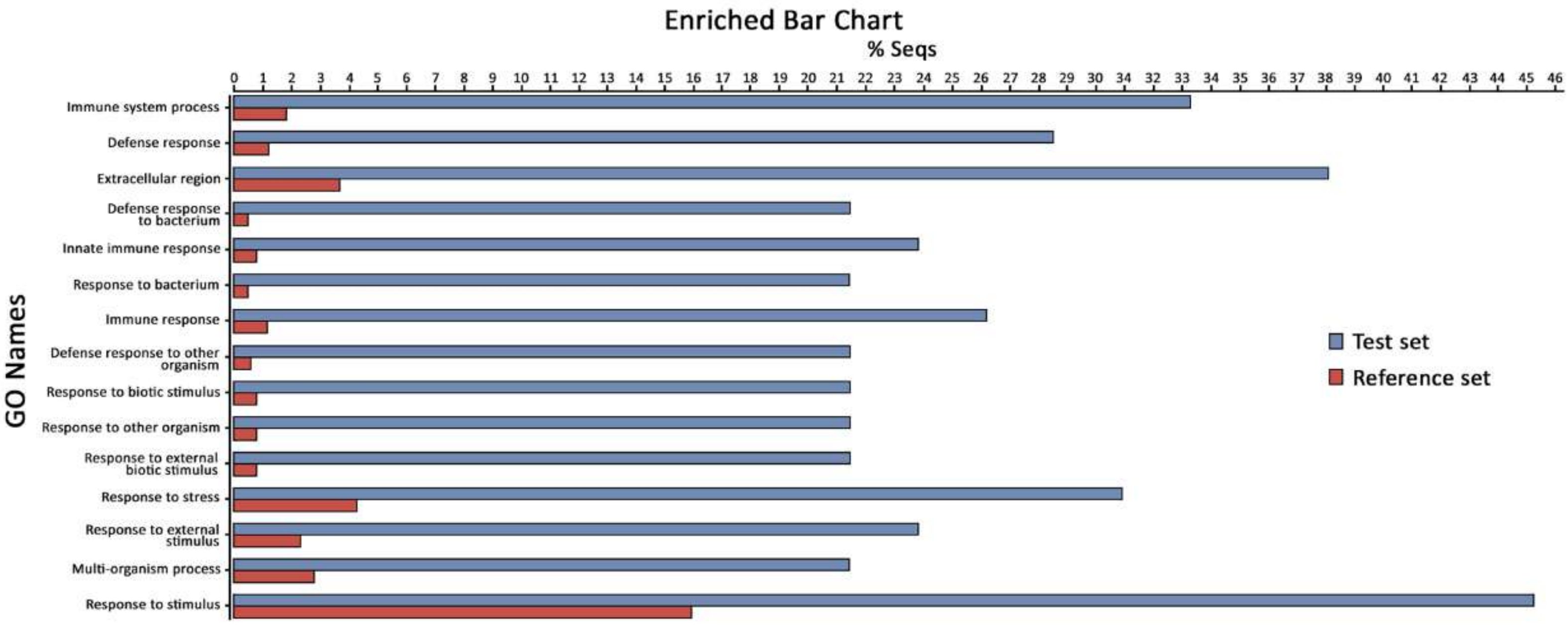

Enriched Bar Chart
% Seqs
0
1
2
3
4
5
6
7
8
9
10
11
12
13
14
15
16
17
18
19
20
21
22
23
24
25
26
27
28
29
30
31
32
33
34
35
36
37
38
39
40
41
42
43
44
45
46
GO Names
Immune system process
Defense response
Extracellular region
Defense response to bacterium
Innate immune response
Response to bacterium
Immune response
Defense response to other organism
Response to biotic stimulus
Response to other organism
Response to external biotic stimulus
Response to stress
Response to external stimulus
Multi-organism process
Response to stimulus
Test set
Reference set

S6 Fig

A

B

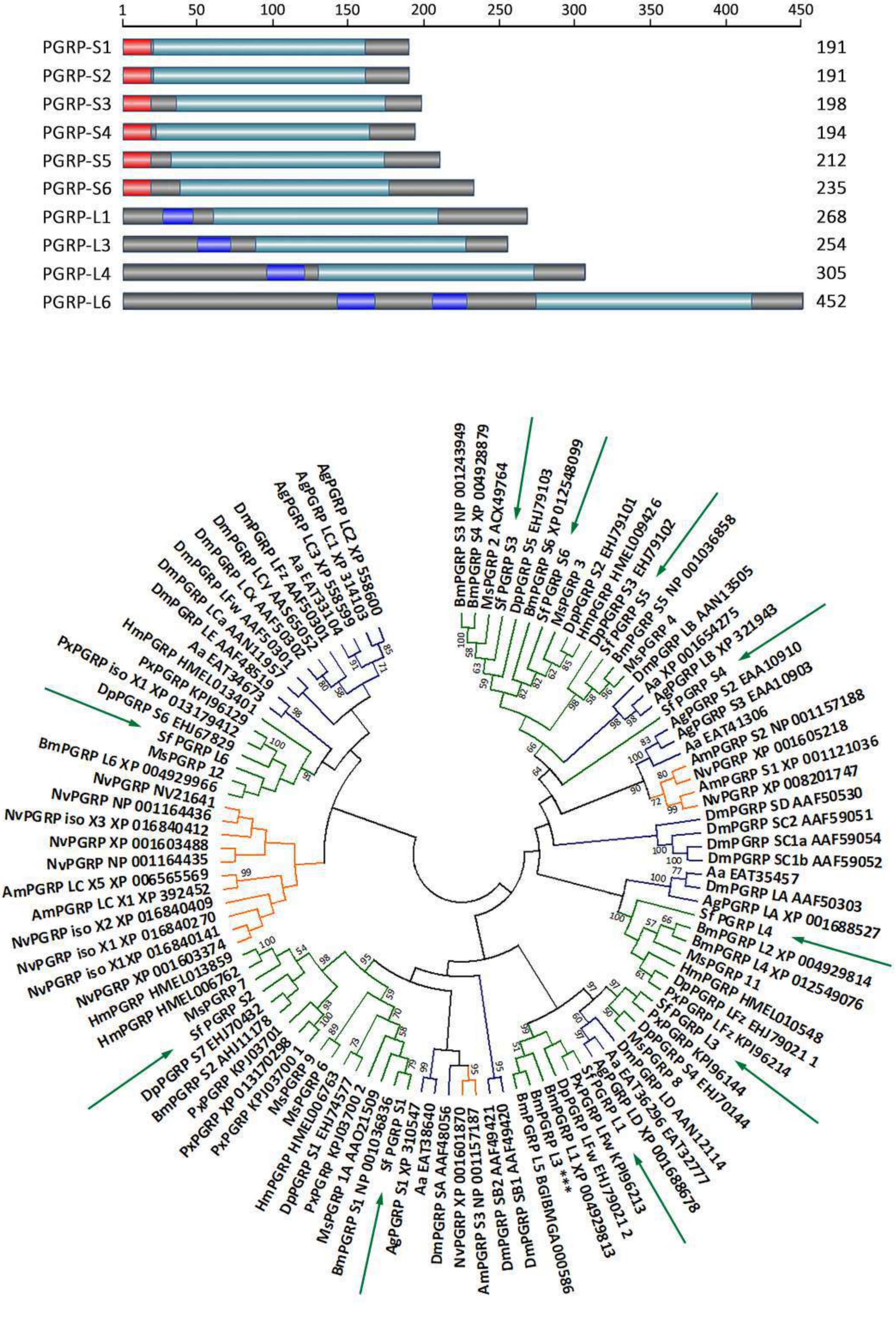

1
50
100
150
200
250
300
350
400
450
PGRP-S1 191
PGRP-S2 191
PGRP-S3 198
PGRP-S4 194
PGRP-S5 212
PGRP-S6 235
PGRP-L1 268
PGRP-L3 254
PGRP-L4 305
PGRP-L6 452
BmPGRP S3 NP 001243949
BmPGRP S4 XP 004928879
MsPGRP 2 ACX49764
Sf PGRP S3
DpPGRP S5 EHJ79103
BmPGRP S6 XP 012548099
Sf PGRP S6
MsPGRP 3
DpPGRP S2 EHJ79101
HmPGRP HMEL009426
DpPGRP S3 EHJ79102
Sf PGRP S5
BmPGRP S5 NP 001036858
MsPGRP 4
DmPGRP LB AAN13505
Aa XP 001654275
AgPGRP LB XP 321943
Sf PGRP S4
AgPGRP S2 EAA10910
AgPGRP S3 EAA10903
Aa EAT41306
AmPGRP S2 NP 001157188
NvPGRP XP 001605218
AmPGRP S1 XP 001121036
NvPGRP XP 008201747
DmPGRP SD AAF50530
DmPGRP SC2 AAF59051
DmPGRP SC1a AAF59054
DmPGRP SC1b AAF59052
Aa EAT35457
DmPGRP LA AAF50303
AgPGRP LA XP 001688527
Sf PGRP L4
BmPGRP L2 XP 004929814
BmPGRP L4 XP 012549076
MsPGRP 11
HmPGRP HMEL010548
DpPGRP LFz EHJ79021 1
PxPGRP LFz KPI96214
Sf PGRP L3
PxPGRP KPI96144
DpPGRP S4 EHJ70144
MsPGRP 8
DmPGRP LD AAN12114
Aa EAT36296 EAT32777
AgPGRP LD XP 001688678
Sf PGRP L1
PxPGRP LFw KPI96213
DpPGRP LFw EHJ79021 2
BmPGRP L1 XP 004929813
BmPGRP L3 ***
BmPGRP L5 BGIBMGA000586
DmPGRP SB1 AAF49420
DmPGRP SB2 AAF49421
AmPGRP S3 NP 001157187
NvPGRP XP 001601870
DmPGRP SA AAF48056
Aa EAT38640
AgPGRP S1 XP 310547
Sf PGRP S1
BmPGRP S1 NP 001036836
MsPGRP 1A AAO21509
PxPGRP KPJ03700 2
DpPGRP S1 EHJ74577
HmPGRP HMEL006763
MsPGRP 6
MsPGRP 9
PxPGRP KPJ03700 1
PxPGRP XP 013170298
PxPGRP KPJ03701
BmPGRP S2 AHJ11178
DpPGRP S7 EHJ70432
Sf PGRP S2
MsPGRP 7
HmPGRP HMEL006762
HmPGRP HMEL013859
NvPGRP XP 001603374
NvPGRP iso X1XP 016840141
NvPGRP iso X1 XP 016840270
NvPGRP iso X2 XP 016840409
AmPGRP LC X1 XP 392452
AmPGRP LC X5 XP 006565569
NvPGRP NP 001164435
NvPGRP XP 001603488
NvPGRP iso X3 XP 016840412
NvPGRP NP 001164436
NvPGRP NV21641
BmPGRP L6 XP 004929966
MsPGRP 12
Sf PGRP L6
DpPGRP S6 EHJ67829
PxPGRP iso X1 XP 013179412
PxPGRP KPI96129
HmPGRP HMEL013401
Aa EAT34673
DmPGRP LE AAF48519
DmPGRP LCa AAN11957
DmPGRP LFw AAF50301
DmPGRP LCx AAF50302
DmPGRP LCy AAS65052
DmPGRP LFz AAF50301
Aa EAT33104
AgPGRP LC3 XP 558599
AgPGRP LC1 XP 314103
AgPGRP LC2 XP 558600

# S7 Fig

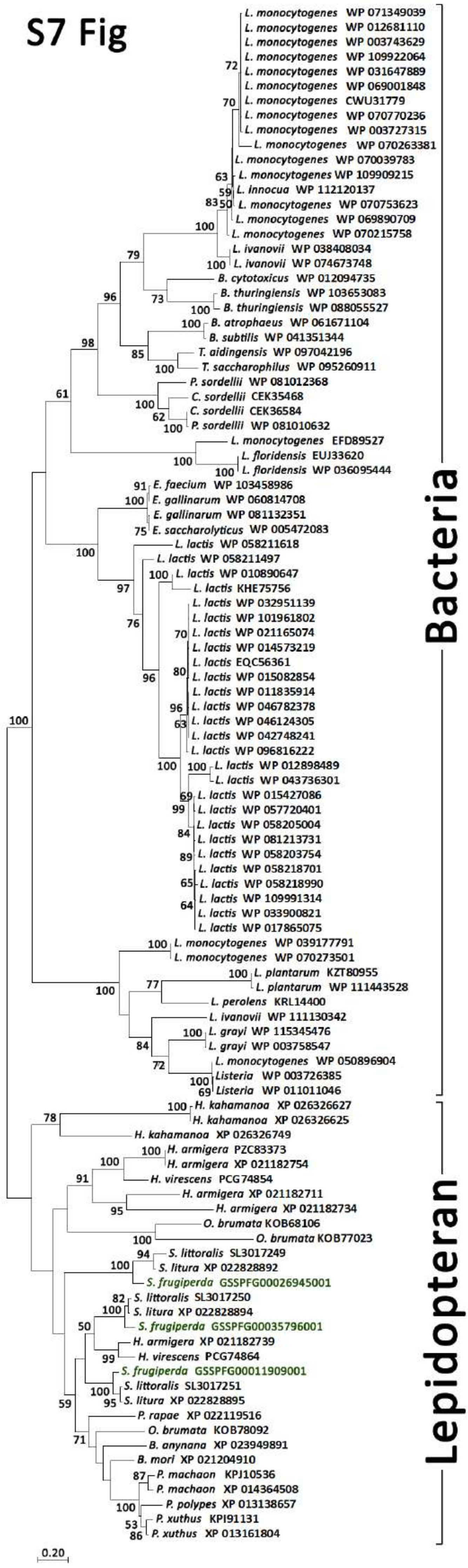
L. monocytogenes WP 071349039
L. monocytogenes WP 012681110
L. monocytogenes WP 003743629
L. monocytogenes WP 109922064
L. monocytogenes WP 031647889
L. monocytogenes WP 069001848
L. monocytogenes CWU31779
L. monocytogenes WP 070770236
L. monocytogenes WP 003727315
L. monocytogenes WP 070263381
L. monocytogenes WP 070039783
L. monocytogenes WP 109909215
L. innocua WP 112120137
L. monocytogenes WP 070753623
L. monocytogenes WP 069890709
L. monocytogenes WP 070215758
L. ivanovii WP 038408034
L. ivanovii WP 074673748
B. cytotoxicus WP 012094735
B. thuringiensis WP 103653083
B. thuringiensis WP 088055527
B. atrophaeus WP 061671104
B. subtilis WP 041351344
T. aidingensis WP 097042196
T. saccharophilus WP 095260911
P. sordellii WP 081012368
C. sordellii CEK35468
C. sordellii CEK36584
P. sordellii WP 081010632
L. monocytogenes EFD89527
L. floridensis EUJ33620
L. floridensis WP 036095444
E. faecium WP 103458986
E. gallinarum WP 060814708
E. gallinarum WP 081132351
E. saccharolyticus WP 005472083
L. lactis WP 058211618
L. lactis WP 058211497
L. lactis WP 010890647
L. lactis KHE75756
L. lactis WP 032951139
L. lactis WP 101961802
L. lactis WP 021165074
L. lactis WP 014573219
L. lactis EQC56361
L. lactis WP 015082854
L. lactis WP 011835914
L. lactis WP 046782378
L. lactis WP 046124305
L. lactis WP 042748241
L. lactis WP 096816222
L. lactis WP 012898489
L. lactis WP 043736301
L. lactis WP 015427086
L. lactis WP 057720401
L. lactis WP 058205004
L. lactis WP 081213731
L. lactis WP 058203754
L. lactis WP 058218701
L. lactis WP 058218990
L. lactis WP 109991314
L. lactis WP 033900821
L. lactis WP 017865075
L. monocytogenes WP 039177791
L. monocytogenes WP 070273501
L. plantarum KZT80955
L. plantarum WP 111443528
L. perolens KRL14400
L. ivanovii WP 111130342
L. grayi WP 115345476
L. grayi WP 003758547
L. monocytogenes WP 050896904
Listeria WP 003726385
Listeria WP 011011046
H. kahamanoa XP 026326627
H. kahamanoa XP 026326625
H. kahamanoa XP 026326749
H. armigera PZC83373
H. armigera XP 021182754
H. virescens PCG74854
H. armigera XP 021182711
H. armigera XP 021182734
O. brumata KOB68106
O. brumata KOB77023
S. littoralis SL3017249
S. litura XP 022828892
S. frugiperda GSSPFG00026945001
S. littoralis SL3017250
S. litura XP 022828894
S. frugiperda GSSPFG00035796001
H. armigera XP 021182739
H. virescens PCG74864
S. frugiperda GSSPFG00011909001
S. littoralis SL3017251
S. litura XP 022828895
P. rapae XP 022119516
O. brumata KOB78092
B. anynana XP 023949891
B. mori XP 021204910
P. machaon KPJ10536
P. machaon XP 014364508
P. polypes XP 013138657
P. xuthus KPI91131
P. xuthus XP 013161804
Bacteria
Lepidopteran
0.20
